# The Contextual Essentiality of Mitochondrial Genes in Cancer

**DOI:** 10.3389/fcell.2021.695351

**Published:** 2021-10-22

**Authors:** Luke W. Thomas, Margaret Ashcroft

**Affiliations:** Department of Medicine, University of Cambridge, Cambridge, United Kingdom

**Keywords:** mitochondria, essentiality, metabolism, viability, signaling

## Abstract

Mitochondria are key organelles in eukaryotic evolution that perform crucial roles as metabolic and cellular signaling hubs. Mitochondrial function and dysfunction are associated with a range of diseases, including cancer. Mitochondria support cancer cell proliferation through biosynthetic reactions and their role in signaling, and can also promote tumorigenesis via processes such as the production of reactive oxygen species (ROS). The advent of (nuclear) genome-wide CRISPR-Cas9 deletion screens has provided gene-level resolution of the requirement of nuclear-encoded mitochondrial genes (NEMGs) for cancer cell viability (essentiality). More recently, it has become apparent that the essentiality of NEMGs is highly dependent on the cancer cell context. In particular, key tumor microenvironmental factors such as hypoxia, and changes in nutrient (e.g., glucose) availability, significantly influence the essentiality of NEMGs. In this mini-review we will discuss recent advances in our understanding of the contribution of NEMGs to cancer from CRISPR-Cas9 deletion screens, and discuss emerging concepts surrounding the context-dependent nature of mitochondrial gene essentiality.

## Mitochondria and Cancer

Mitochondria are thought to have arisen from the endosymbiotic fusion of a protobacterium that had evolved the ability to generate chemical energy through oxidative phosphorylation (OXPHOS), and an archaeal host cell (Andersson et al., 1998; Fitzpatrick et al., 2006). Over evolutionary time, mitochondria have become centrally embedded in eukaryotic cell biology, from a physical, genetic, and functional point of view. Indeed, mitochondria now play host to an array of biochemical reactions and pathways that support cell viability, and influence survival beyond the bioenergetic role of OXPHOS. In cancer, the preponderance of studies show that many of the metabolic pathways and metabolites found in mitochondria play a central role in promoting tumor growth and disease progression. For example, it has been shown that OXPHOS-dependent mitochondrial metabolism is pivotal for the availability of amino acids to support biomass accumulation in proliferating cells, particularly the synthesis of aspartate (Birsoy et al., 2015; Sullivan et al., 2015). Moreover, mitochondria are important sources of precursors for nucleotide synthesis, such as folate, which is required for *de novo* purine and pyrimidine synthesis pathways (Garrow et al., 1993; Morscher et al., 2018). In addition, the mitochondrial IMS is the site of an OXPHOS-linked step in the uridine salvage pathway (Loffler et al., 1997), and key components of the redox-regulated import disulfide relay system within the mitochondrial IMS have been shown to regulate tumor growth (Yang et al., 2012; Hangen et al., 2015; Al-Habib and Ashcroft, 2021). Furthermore, lipid metabolism is dependent on mitochondrial function, both through catabolism to generate ATP via β-oxidation of fatty acids (Houten and Wanders, 2010), as well as anabolism to generate precursors of fatty acids (DeBerardinis et al., 2007; Wise et al., 2011), steroids (Miller, 2011), and phospholipids (Osman et al., 2011). Aside from the synthesis of ATP and precursors for biomass accumulation, mitochondria are the site of many other biochemical pathways that are critical for supporting tumor cell viability, such as the detoxification of exogenous bioactive molecules, heme biosynthesis, and carbonic acid metabolism (Thomas et al., 2021). Mitochondria as key signaling organelles, provide important regulation of calcium homeostasis (Giorgi et al., 2018), and are a source of ROS generation (Shadel and Horvath, 2015), both of which are known to influence a range of disease phenotypes in cancer (Sabharwal and Schumacker, 2014; Liu et al., 2020; Perillo et al., 2020). Beyond the role of mitochondria in supporting cancer cell proliferation, upregulation of mitochondrial biogenesis has been identified as a key driver of invasion and metastasis in breast cancer (LeBleu et al., 2014). Along with performing essential functions that support proliferation and survival, mitochondria are also involved in promoting several distinct forms of cell death. These include apoptosis, involving the cytoplasmic release of molecules from the mitochondrial intermembrane space (IMS) (Singh et al., 2019), and ferroptosis, involving iron-dependent lipid peroxidation (Conrad et al., 2021). Mitochondrially-mediated apoptosis plays a fundamental role in embryonic development (Ke et al., 2018), as well as in the removal of damaged (Aubrey et al., 2018) or unwanted (Perez-Figueroa et al., 2021) cells throughout the lifespan of humans. Conversely, resistance to mitochondrial apoptosis is a hallmark of cancer (Hanahan and Weinberg, 2011), and an important mediator of treatment resistance (Carneiro and El-Deiry, 2020).

There are important tissue-specific and pathway-specific differences in the degree to which mitochondrial functions contribute to cancer. For example, *in silico* analysis of transcriptomic data from multiple tumor types identified that mitochondrial OXPHOS genes were upregulated in 35% of cancer types, but downregulated in a further 25% (Gaude and Frezza, 2016), including clear cell renal cell carcinoma (ccRCC). Indeed, the downregulation of oxygen consumption rate (OCR) and OXPHOS is a well-characterized feature of *VHL* inactivated ccRCC cells, and is partially buffered by the metabolic rewiring of ccRCC cells by constitutive activation of the hypoxia-inducible factor (HIF) pathway (Nilsson et al., 2015; Briston et al., 2018). Furthermore, mutations in the TCA cycle genes *IDH1/2, FH*, and subunits A-D of succinate dehydrogenase (SDH), are driver mutations in certain cancers, including renal cancers and gliomas (Han et al., 2020; Yong et al., 2020). Interestingly, mutations in these genes leads to the intracellular accumulation of oncometabolites (i.e., 2-hydroxyglutarate, fumarate, and succinate), which drive disease progression through their effects on multiple pathways, such as DNA methylation and the HIF pathway (Han et al., 2020; Yong et al., 2020).

Thus, while normal mitochondrial functions can support the development and progression of many cancers, these effects are not universal, either across cancers or across mitochondrial functions. A comprehensive review of the contributions of different mitochondrial functions to cancer is beyond the scope of this mini-review, and can be found elsewhere (Wallace, 2012; Vyas et al., 2016).

## Essential Mitochondrial Genes

Much has been uncovered regarding the importance of mitochondria to cancer biology in small-scale (single gene/cancer) studies (reviewed in Wallace, 2012; Vyas et al., 2016). However, it is clear that both the multi-gene complexity of mitochondrial metabolism, and the importance of interactions between mitochondria and the cellular context (e.g., oncogenic mutations, microenvironmental conditions) necessitate more comprehensive efforts to fully understand the importance of mitochondrial genes in cancer. The advent of genome-wide CRISPR-Cas9 deletion screening techniques (Evers et al., 2016) has allowed such comprehensive identification of NEMGs that are absolutely required for the viability of cultured cancer cells—genes which can therefore be described as essential. It should be noted however that, as yet, the published genome-wide CRISPR-Cas9 single guide (sg)RNA libraries do not contain sgRNAs that target genes within the mitochondrial genome (mtDNA). CRISPR-Cas9 targeting of mtDNA has been challenging due to a lack of recognition sites for the Cas9 enzyme, and the poor delivery of sgRNA into mitochondria. However, recent studies have described the generation of gene-editing systems that can be used for targeting mitochondrially-encoded mitochondrial genes (MEMGs) (Mok et al., 2020; Hussain et al., 2021). It is clear from both ethidium bromide and enzymatic depletion of mtDNA in cancer cells, that MEMGs are also required for the proliferation of many cancer cell lines (Herst et al., 2004; Kukat et al., 2008). Several studies have investigated the mutation landscape of mtDNA in cancer, and have found that loss-of-function mtDNA mutations are among the most frequent genetic events in cancer cells, at a rate comparable to many common cancer driver genes (Gorelick et al., 2021). Interestingly, however, these mutations were found in two independent studies to be enriched in subunits of respiratory complex (C)I, but depleted from subunits of CV (ATPase), suggesting differing contributions of each respiratory complex to cancer cell viability (Yuan et al., 2020; Gorelick et al., 2021). Furthermore, loss-of-function mutations in mtDNA-encoded CI genes were found to be specifically enriched in cancers of the kidney, colon, and thyroid (Hopkins et al., 2017; Yuan et al., 2020; Gorelick et al., 2021), highlighting that tissue etiology is a central determinant of the importance of mitochondria to cancer development and progression.

**FIGURE 1:**
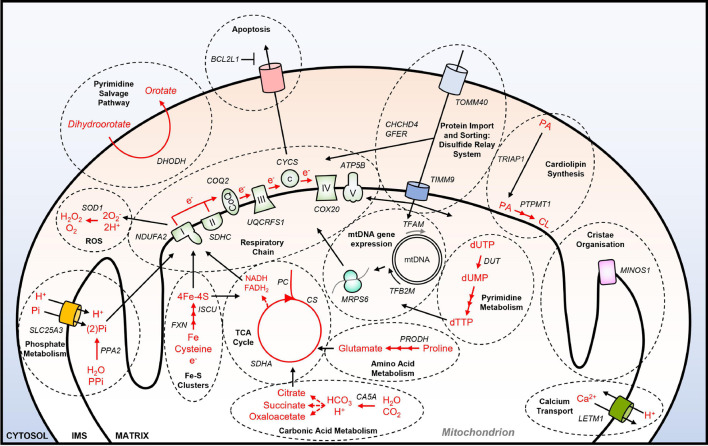
Essential NEMGs and their mitochondrial pathway function. Model shows selected NEMGs identified as common essential genes by the DepMap project, and other genome-wide CRISPR-Cas9 deletion screens of cancer cell lines (Arroyo et al., 2016; Tsherniak et al., 2017; Bao et al., 2021; Thomas et al., 2021). For a more comprehensive list of common essential NEMGs (see Thomas et al., 2021). Selected common essential NEMGs are denoted in capital letters and italics. Black arrows denote transport/movement/relationship. Red arrows and red text denote biochemical reactions. Multiple arrow heads denote multiple reactions. I, respiratory complex I; II, respiratory complex II; III, respiratory complex III, IV, respiratory complex IV; V, respiratory complex V; CoQ, Coenzyme Q; c, cytochrome c; PA, phosphatidic acid; CL, cardiolipin; mtDNA, mitochondrial DNA; IMS, intermembrane space; ROS; reactive oxygen species; PPi, inorganic pyrophosphate.

A systematic effort to identify genetic dependencies in cancer cell lines under standard culture conditions is being carried out by the Achilles project, also known as the Dependency Map (DepMap) consortium (Tsherniak et al., 2017). Using data from this project, a functional annotation of all 1158 nuclear-encoded genes that encode mitochondrially-localized proteins (Calvo et al., 2016) has been performed recently (Thomas et al., 2021). These analyses identified that 23.2% (264) of NEMGs are essential for the growth of over 90% of the 625 tumor cell lines tested by DepMap, which are therefore referred to as common essential genes. It was also shown that NEMGs are enriched for common essential genes relative to the genome as a whole (Thomas et al., 2021). Functional annotation of the 264 essential NEMGs confirmed many of the previously identified genes and pathways that are important for cancer cell viability (as outlined in section “Mitochondria and Cancer”) (Figure 1). These include genes involved in: OXPHOS (e.g., *NDUFA2*, *CYCS*); the TCA cycle (e.g., *SDHC*, *PC*); lipid metabolism (e.g., *ABCB10*, *PTPM1*); nucleotide metabolism (e.g., *DHODH*); amino acid metabolism (e.g., *PRODH*, *PRODH2*); calcium transport (e.g., *LETM1*); ROS production and detoxification (e.g., *ROMO1*, *SOD1*); apoptosis (e.g., *BCL2L1*). Furthermore, common essential NEMGs were also identified from pathways that have not been previously characterized as being required for the viability of cancer cells, such as carbonic acid metabolism (*CA5A*), and phosphate metabolism (*PPA2*, *SLC25A3*) (Figure 1).

Many of these essential NEMGs have also been identified in other studies where single cell line genome-wide CRISPR-Cas9 deletion screens have been undertaken, using different cancer cell lines (Arroyo et al., 2016; Jain et al., 2016; Bao et al., 2021; Niu et al., 2021; Thomas et al., 2021). Interestingly, while there is broad pathway-level agreement between these independent studies, there is little gene-to-gene agreement, and in some cases, gene hits identified from the CRISPR-Cas9 deletion screen could not be verified in validation experiments using the primary screening cell line or additional cell lines (Jain et al., 2020). Thus, while CRISPR-Cas9 deletion screens of this kind are powerful tools in determining gene essentiality, care must be taken when comparing between studies, due to factors such as the methodological differences in culturing conditions, the sgRNA library used, and the cell line(s) selected for the screen. Indeed, the DepMap study clearly demonstrates that specific gene-to-gene essentiality—including NEMGs—can vary greatly across cancer cell lines, and even between cell lines with similar tissue etiologies (Tsherniak et al., 2017). Beyond the importance of genetic context to the essentiality of NEMGs and pathways, the metabolic environment has also been shown to significantly influence mitochondrial gene essentiality, using CRISPR-Cas9 deletion screens.

## Mitochondrial Gene Essentiality and Microenvironmental Context

The DepMap project (Tsherniak et al., 2017), and other independent genome-wide CRISPR-Cas9 deletion screens (e.g., To et al., 2019), constitute powerful resources for understanding gene essentiality in cancer cells. However, only a few screens use culture conditions that reflect features of the (solid) tumor microenvironment (TME) (Arroyo et al., 2016; Jain et al., 2020; Bao et al., 2021; Niu et al., 2021; Thomas et al., 2021). The TME is central to promoting tumorigenesis and disease progression, and comprises features that are distinct from surrounding normal tissue (Egeblad et al., 2010; Elia and Haigis, 2021). Along with tumor cells, the TME is a multicellular compartment which includes infiltrating immune cells, has high extracellular matrix density, and poor vascular perfusion which limits both nutrient (e.g., glucose) and oxygen availability. Low oxygen levels, known as hypoxia, is a common feature of the TME, and is associated with treatment resistance (Begg and Tavassoli, 2020), as well as poor prognosis in patients (Choudhry and Harris, 2018). As the major site of oxygen consumption in the cell, mitochondria are profoundly influenced by the availability of cellular oxygen, and carbon sources such as glucose, which provide reducing equivalents for OXPHOS (Thomas and Ashcroft, 2019).

**FIGURE 2:**
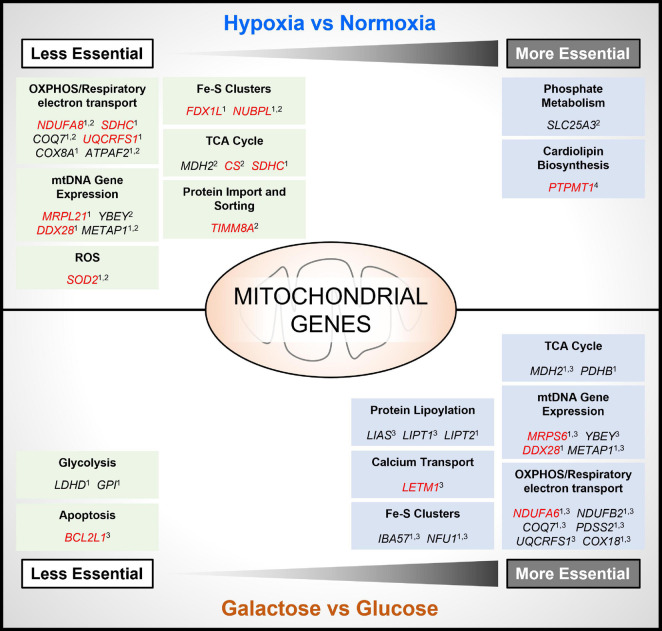
Contextual essentiality of mitochondrial genes. Model shows NEMGs identified from genome-wide CRISPR-Cas9 deletion screens of cancer cell lines, shown to be selectively essential under specific cancer cell culture conditions [hypoxia, normoxia, glucose and glucose-free (galactose)]. Studies included are indicated as follows: 1 (Thomas et al., 2021); 2 (Jain et al., 2020); 3 (Arroyo et al., 2016); 4 (Bao et al., 2021). Red highlighted genes were identified as common essential under standard culture conditions by the DepMap Project (Tsherniak et al., 2017).

To mimic conditions of the TME, several recent genome-wide CRISPR-Cas9 deletion screens have been carried out under conditions of hypoxia (Jain et al., 2020; Bao et al., 2021; Niu et al., 2021; Thomas et al., 2021), or glucose deprivation (Arroyo et al., 2016; Thomas et al., 2021), using different cancer cell types. Importantly, while there are differences in the essentiality of specific genes identified between studies, there is broad agreement in the findings across these studies showing that the essentiality of both non-mitochondrial and mitochondrial genes is dependent on the environmental context in which cells are cultured (Figure 2). For example, in two studies it was found that the deletion of numerous genes involved in OXPHOS improved the viability of tumor cells cultured in hypoxia (Jain et al., 2020; Thomas et al., 2021). Interestingly, these genes included a subset of genes that were found to be essential for tumor cell viability under standard culture conditions (normoxia and glucose), such as subunits of respiratory complexes complex I (e.g., *NDUFA8*) and complex II (e.g., *SDHC*), and enzymes involved in the ubiquinone synthesis pathway (e.g., *COQ7*), or clearing mitochondrial ROS (e.g., *SOD2*) (Figure 2). These findings are perhaps not surprising, as a reduction in overall OCR, and a suppression of mitochondrial biogenesis and protein expression are well-characterized cellular responses to hypoxia, that are mediated in part through the HIF pathway (Thomas and Ashcroft, 2019).

Moreover, in the study by Bao and colleagues, the gene *PTPMT1*—a NEMG that is required for synthesis of the essential mitochondrial membrane lipid cardiolipin (Figure 1)—was discovered to become significantly more essential for the growth of MHCC97L hepatocellular carcinoma (HCC) cells in hypoxia (Bao et al., 2021; Figure 2). Interestingly, deletion of *PTPMT1* decreased cellular cardiolipin content, and led to significantly increased intracellular ROS levels in hypoxia (Bao et al., 2021). Thus, mitochondrial cardiolipin synthesis appears to support HCC cell survival in hypoxia, through mitigation of the cytotoxic effects of ROS production, which is elevated in hypoxia (Thomas and Ashcroft, 2019; Bao et al., 2021). Notably however, *PTPMT1* was identified as a common essential gene in normoxia (Tsherniak et al., 2017; Thomas et al., 2021), and was not found to be significantly more or less essential in other hypoxia CRISPR-Cas9 deletion screens (Jain et al., 2020; Thomas et al., 2021). This again highlights that mitochondrial (and non-mitochondrial) gene essentiality is also dependent on cell type, and methodological differences between CRISPR-Cas9 deletion studies must be taken into account when comparing datasets. Related to this, while the genes encoding the HIF pathway proteins HIF-1α (*HIF1A*) and HIF1-β (*ARNT*) were found to be the two most essential for hypoxic survival in MHCC97L (HCC) cells (Bao et al., 2021), no HIF pathway genes were among the most essential genes identified from CRISPR-Cas9 deletion screens using K562 chronic myeloid leukemia cells (Jain et al., 2020), or U2OS osteosarcoma cells (Thomas et al., 2021).

While specific NEMGs were shown to be less essential in hypoxia compared to normoxia, in contrast their essentiality increased in cells cultured in the absence of glucose (and substitution with galactose) (Arroyo et al., 2016; Thomas et al., 2021; Figure 2). Galactose, like glucose, can be metabolized to pyruvate to support OXPHOS. Unlike the metabolism of glucose to pyruvate in glycolysis, metabolism of galactose to pyruvate produces no net ATP (Rossignol et al., 2004), and increases the reliance of cells on OXPHOS for bioenergetic homeostasis, and thus cells become more sensitive to inhibitors of the respiratory chain (Marroquin et al., 2007). In agreement, a large number of the differentially essential genes identified in both the Arroyo et al. and Thomas et al. independent genome-wide CRISPR deletion screens in galactose, were found to be genes that encode respiratory complex subunits (e.g., *NDUFA6*), and OXPHOS accessory proteins, such as those involved in Fe/S cluster formation (e.g., *IBA57*, *NFU1*), and the ubiquinone synthesis pathway (*COQ7*) (Figure 2). Another large subset of essential NEMGs in galactose were found to be related to maintenance and regulation of expression of genes from mtDNA, such as *MRPS6* and *YBEY* (Figure 2). Beyond OXPHOS, NEMGs from numerous other mitochondrial pathways were also more essential in galactose in both studies (Arroyo et al., 2016; Thomas et al., 2021), including genes involved in fatty acid metabolism (*MCAT*) and the TCA cycle (e.g., *MDH2*). These studies clearly highlight the importance of the metabolic context for mitochondrial gene essentiality in cancer cells, but it should be noted that galactose substitution of glucose in culture may not entirely recapitulate the effects of glucose deprivation found in the TME or fully mimic the effects of metabolic reprogramming of glucose in cancer cells (Hay, 2016).

## Therapeutic Implications

In cancer, hypoxia and dysregulated mitochondrial metabolism are key features of (solid) tumors, and are associated with metabolic reprogramming and disease progression (Weinberg and Chandel, 2015; Thomas and Ashcroft, 2019; Frattaruolo et al., 2020). Tumor cells rely on both glycolysis and OXPHOS to survive, and thus OXPHOS has become an increasingly attractive area for therapeutic exploitation in cancer (Molina et al., 2018; Shi et al., 2019; Vasan et al., 2020). Of particular interest, from a safety perspective, is the use of drug repurposing which has identified OXPHOS inhibitors with anti-tumor activity (Pernicova and Korbonits, 2014; Ashton et al., 2016; Aminzadeh-Gohari et al., 2020; Zhang et al., 2020). Furthermore, while the lethality of glucose deprivation for many cultured cancer cells constitutes a significant technical challenge when carrying out CRISPR-Cas9 deletion screens under this condition, the findings from such screens (Arroyo et al., 2016; Thomas et al., 2021) provide valuable insight into targeting glucose metabolism in cancer as a potential therapeutic strategy (Hay, 2016). Given the importance of mitochondria for normal physiological processes, delineating how mitochondrial gene function underlies tumorigenesis, and in the context of hypoxia, will be vital for understanding the potential therapeutic benefit of exploiting mitochondrial function(s) in cancer.

Finally, outwith cancer, the findings from CRISPR-Cas9 deletion screens and other studies (Arroyo et al., 2016; Jain et al., 2016; Thomas et al., 2021) have important therapeutic implications for mitochondrial disease, as they show that hypoxia promotes survival and can protect cells from the potentially deleterious effects of mitochondrial gene mutation and loss of function. Whether hypoxia could provide therapeutic benefit clinically in mitochondrial disease (Jain et al., 2016), or offer a means to strategically reveal drug-induced mitochondrial toxicity of novel therapeutic agents as part of the preclinical drug development path, is of intense interest and has yet to be realized.

## Conclusion

Mitochondria are key metabolic and signaling hubs in eukaryotic cells, and are the site of numerous biochemical pathways that support cell viability. A growing body of evidence has demonstrated that the importance of mitochondrial functions for cell viability can vary depending on the genetic and metabolic context. Recently, genome-wide CRISPR-Cas9 deletion screens have allowed for the identification of individual mitochondrial (and non-mitochondrial) genes that are essential for the viability of cancer cell lines, under different tumor-relevant conditions. These approaches have revealed important insights into the contextual nature of mitochondrial gene essentiality in cancer cells, and provide a unique resource of potential therapeutic targets for further investigation and exploitation. Interestingly, genome-wide CRISPR screens are also being applied for target deconvolution in drug development studies (Neggers et al., 2018), and their future applications may allow for the identification of genes underlying disease-relevant phenotypes other than viability (e.g., migration).

## Author Contributions

LWT and MA wrote the manuscript. MA secured the funding. Both authors contributed to the article and approved the submitted version.

## Conflict of Interest

The authors declare that the research was conducted in the absence of any commercial or financial relationships that could be construed as a potential conflict of interest.

## Publisher’s Note

All claims expressed in this article are solely those of the authors and do not necessarily represent those of their affiliated organizations, or those of the publisher, the editors and the reviewers. Any product that may be evaluated in this article, or claim that may be made by its manufacturer, is not guaranteed or endorsed by the publisher.
